# Adaptive Potential of the Heme Oxygenase/Carbon Monoxide Pathway During Hypoxia

**DOI:** 10.3389/fphys.2020.00886

**Published:** 2020-07-22

**Authors:** Michael S. Tift, Rodrigo W. Alves de Souza, Janick Weber, Erica C. Heinrich, Francisco C. Villafuerte, Atul Malhotra, Leo E. Otterbein, Tatum S. Simonson

**Affiliations:** ^1^Department of Biology and Marine Biology, University of North Carolina Wilmington, Wilmington, NC, United States; ^2^Department of Surgery, Beth Israel Deaconess Medical Center and Harvard Medical School, Boston, MA, United States; ^3^Division of Biomedical Sciences, University of California Riverside, School of Medicine, Riverside, CA, United States; ^4^Laboratorio de Fisiología Comparada, Facultad de Ciencias y Filosofía, Universidad Peruana Cayetano Heredia, Lima, Peru; ^5^Division of Pulmonary, Critical Care, and Sleep Medicine, University of California San Diego, School of Medicine, San Diego, CA, United States

**Keywords:** carbon monoxide, heme oxygenase, hypoxia, altitude, diving, evolution, cytoprotection

## Abstract

Heme oxygenase (HO) enzymes catalyze heme into biliverdin, releasing carbon monoxide (CO) and iron into circulation. These byproducts of heme degradation can have potent cytoprotective effects in the face of stressors such as hypoxia and ischemia-reperfusion events. The potential for exogenous use of CO as a therapeutic agent has received increasing attention throughout the past few decades. Further, HO and CO are noted as putatively adaptive in diving mammals and certain high-altitude human populations that are frequently exposed to hypoxia and/or ischemia-reperfusion events, suggesting that HO and endogenous CO afford an evolutionary advantage for hypoxia tolerance and are critical in cell survival and injury avoidance. Our goal is to describe the importance of examining HO and CO in several systems, the physiological links, and the genetic factors that underlie variation in the HO/CO pathway. Finally, we emphasize the ways in which evolutionary perspectives may enhance our understanding of the HO/CO pathway in the context of diverse clinical settings.

## Introduction

In the late 1990s, a concept emerged suggesting that all cells possessed protective genes with the sole purpose to ensure survival (Platt and Nath, 1998). These cytoprotective genes played a role in the production of antioxidants, acute phase proteins, as well as regulating the effects of aging. A few were clearly master regulators, such that when not present, the cell or tissue was sensitive to stress. One of these was *Hmox*, which encodes the protein heme oxygenase (HO), the rate limiting enzyme responsible for the degradation of heme. HO catabolizes heme into biliverdin, which is subsequently converted to bilirubin by biliverdin reductase, releasing carbon monoxide (CO) and iron as byproducts. Paradoxically, CO was strictly considered a toxic pollutant. This notion stems from CO having a high affinity for the O_2_-binding site on hemoproteins. Therefore, elevated levels of CO can reduce O_2_ storage capacity and inhibit O_2_ transport, resulting in CO toxicity. However, recent studies have revealed the biological effects of the gas, leading to the acceptance of CO having therapeutic and cytoprotective effects in modulating inflammation, cell death, and proliferation (Motterlini and Otterbein, 2010).

Heme oxygenase genes are highly conserved and found in most living organisms (Wilks, 2002; Li and Stocker, 2009). The inducible HO-1 and constitutive HO-2 are ~40% identical in their amino acid sequence in humans (Cruse and Maines, 1988), with HO-1 observed at highest levels in the spleen, lung, and visceral adipose and HO-2 in the testes, heart, brain, and stomach (GTEx, V6). Stressors known to stimulate HO-1 expression include oxidative stress, hypoxia, cytokines, heavy metals, and bacterial endotoxins (Abraham, 2002). HO enzymes function as a crucial mechanism to recycle iron in the body through the degradation of heme (Wilks, 2002). The majority of extant vertebrates are known to utilize iron-containing hemoproteins (e.g., hemoglobin, myoglobin, neuroglobin, cytochromes, catalase, and peroxidase) as a mechanism to transport electrons in the respiratory chain for the production of ATP, to reduce oxidative stress and/or to increase the storage capacity and delivery of oxygen (O_2_) to cells (Hardison, 1996). Therefore, the ability to recycle iron stores in the body can offer an adaptive advantage if organisms find themselves iron-limited. The regulation of HO enzymes can also influence erythrocyte lifespan. *Hmox1*^−/−^ mice rarely survive beyond the development and those that do have prolonged erythrocyte lifespans, reduced hemoglobin content, and a reduced erythrocyte size that leads to microcytic anemia (Fraser et al., 2015). Moreover, human patients with reduced erythrocyte lifespans display elevated blood and breath CO levels, suggesting increased HO activity (Strocchi et al., 1992).

Recent studies have shown that species and populations adapted to tolerate chronic hypoxia and/or ischemia/reperfusion (I/R) events have upregulated or modified the HO/CO pathway in a manner that could afford beneficial side effects (Herrera et al., 2008; Simonson et al., 2010; Tift et al., 2014). Here, we highlight some of those model species and populations and discuss how such modifications could provide an evolutionary advantage and how information from these studies may be used to learn more about the applications of the HO/CO pathway in the prevention and treatment of certain diseases (Goebel and Wollborn, 2020).

## Evolutionary Insights from Hypoxia-Adapted Populations

The late August Krogh stated that, “for such a large number of problems there will be some animal of choice, or a few such animals, on which it can be most conveniently studied” (Krogh, 1929). Model organisms naturally adapted for life in challenging conditions offer excellent opportunities to understand how extreme phenotypes afford protective advantages that can be directed to improve the treatment and/or the prevention of specific human pathologies (Carey et al., 2012; Carey, 2015). For example, some diving mammals experience dramatic hypoxemia (Meir et al., 2009) and I/R events (Zapol et al., 1979) during their repeated long-duration breath-holds and do not develop injuries from this lifestyle. Species that have adapted to live at high altitude or under other hypoxic conditions (e.g., burrowing) also avoid pathologies associated with exposure to chronic hypoxia (Logan et al., 2020). These natural models offer unique insights into the adaptive mechanisms underlying tolerance to hypoxia and/or I/R injury.

Some deep-diving mammals are known to experience levels of hypoxemia during dives that resemble arterial oxygen saturations of humans breathing ambient air on the summit of Mount Everest (Meir et al., 2009). These deep-divers also appear to be protected from repeated I/R events that occur in a majority of their tissues on a dive-to-dive basis as a result of the “dive response,” characterized by extreme bradycardia and peripheral ischemia to maintain mean arterial blood pressure during breath-holds (Halasz et al., 1974; Zapol et al., 1979; Allen and Vázquez-Medina, 2019). In 1959, Pugh reported levels of CO in the blood of deep-diving Weddell seals from Antarctica that resembled those of cigarette smokers (Pugh, 1959). Since then, Tift et al. (2014) have discovered that deep-diving northern elephant seals also maintain high levels of CO in the blood with carboxyhemoglobin (COHb) levels between 5 and 11% (Tift et al., 2014). While CO was shown to be high in the blood of two deep-diving pinnipeds, the distribution of CO concentrations in the blood of different diving species is still unknown.

The quantity of CO found in the blood of elephant seals is similar to that previously mentioned in laboratory studies, which demonstrate cytoprotective effects from exogenous CO delivery (Nakao et al., 2006). It is hypothesized that the elevated hemoprotein stores seen in deep-diving mammals are the source of the high CO levels, which could be used as a mechanism to reduce the development of tissue injuries from the chronic hypoxemia and I/R events they experience while diving (Tift and Ponganis, 2019). These high CO levels could indicate alterations in the erythrocyte lifespan and HO activity in specific tissues of these animals. Such levels of CO would likely increase the O_2_-binding affinity of hemoglobin, impacting O_2_-delivery mechanics. Considering the low arterial pO_2_ values routinely experienced during breath-holds (<15–20 mmHg), it is possible that a higher hemoglobin-O_2_ affinity due to moderate COHb levels could preserve blood O_2_ stores, increasing O_2_ availability later in the dive (Storz, 2016). Indeed, the Haldane effect suggests that mice exposed to trace amounts of CO will more efficiently acquire O_2_ and survive longer under conditions of severe hypoxia (Roughton and Darling, 1944). Endogenous CO also impacts mitochondrial O_2_ consumption and ATP production, which could contribute to hypometabolism during breath-holds and exposure to hypoxia (D’Amico et al., 2006).

Species adapted to live at high altitude face a different hypoxic challenge, in that the ambient environment has a reduced pO_2_. Certain pathologies may develop due to acute and chronic hypoxia exposure at altitude (e.g., pulmonary and cerebral edema, pulmonary hypertension, and excessive erythrocytosis). Considering hypoxia as a stressor that increases HO-1 activity (Shibahara et al., 2007), the HO/CO pathway is of interest when studying organisms at high altitude. When laboratory rats were brought from an elevation of 1,006 to 3,048 and 4,572 m, their COHb levels increased linearly with altitude from 0.68 to 1.16, and 1.68%, respectively (McGrath, 1992). Similarly, when healthy adult humans were brought from an altitude near sea level to 3,517 m, their COHb levels increased from 0.79 to 0.95% within 20 h (McGrath et al., 1993). When comparing neonatal sheep (lowland species) and llama (highland species) that underwent gestation at high altitude (3,600 m), only sheep developed pulmonary arterial hypertension (Herrera et al., 2008; Llanos et al., 2012). This was associated with reduced soluble guanylate cyclase, HO, and CO production despite increased nitric oxide synthase activity in the lungs of the sheep. In contrast, neonatal llamas avoided pulmonary hypertension and had an increase in pulmonary CO production and HO-1 expression with no change in nitric oxide levels (Herrera et al., 2008). This specific HO/CO protective effect has not been investigated in other species adapted for living with chronic hypoxia.

Humans of Tibetan ancestry exhibit a lower average hemoglobin concentration compared to sojourners and long-term highland residents in the Andes, where individuals exhibit varying degrees of polycythemia (Beall, 2007). Whether relatively lower hemoglobin provides a direct adaptive effect or is the side effect of other traits that provide an advantage remains an important question in the field (Storz, 2010; Simonson 2015). *HMOX2* has been detected as a top selection candidate gene in ours and others’ studies of Tibetan adaptation and is hypothesized to play a role in regulating heme catabolism and CO production (Simonson et al., 2010; Yang et al., 2016). A study of 1,250 high-altitude native Tibetans found variants at this locus that are associated with decreased hemoglobin concentration in males, and *in vitro* analysis indicates that a derived intronic variant (rs4786504) is associated with increased *HMOX2* expression (Yang et al., 2016). While it remains to be determined if regulatory variants at this locus lead to increased heme catabolism, such alterations could contribute to the lower hemoglobin concentrations seen in Tibetans living at altitude.

**Figure 1:**
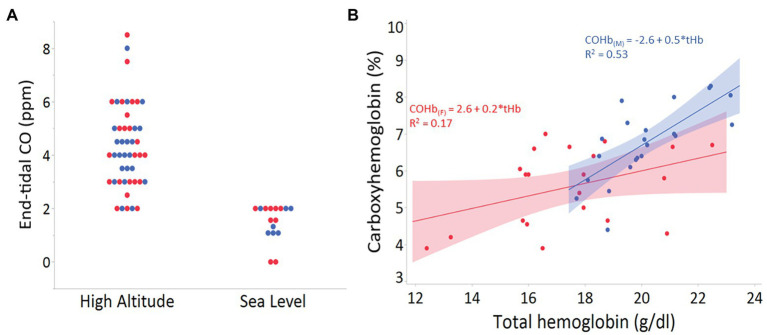
**(A)** End-tidal carbon monoxide (CO) levels (ppm) in 47 adults from Cerro de Pasco, Peru (high altitude), and 18 adults of mixed ancestry living in San Diego, California, USA (sea level). There was no statistical difference between males (blue) and females (red) from high or low altitude groups (*p* > 0.8 for both). **(B)** Relationship between total hemoglobin (g/dl) and the percent carboxyhemoglobin (COHb) in venous blood of adult males (*n* = 22; blue) and females (*n* = 21; red) from Cerro de Pasco, Peru (4,330 m).

In contrast to Tibetans, adaptive *HMOX* genetic variants have not yet been identified in Andean or Ethiopian highlanders. However, HO could mitigate various challenges imposed by hypoxia at altitude (e.g., higher Hb-O_2_ affinity; Storz, 2016). Our recent examination in Andean adult males and females living at 4,350 m shows a positive relationship between endogenous CO in breath and blood with both hemoglobin concentration and altitude (Figure 1). These preliminary findings could reflect elevated erythrocyte destruction, reduced erythrocyte lifespan, and/or increased HO-1 or HO-2 activity. Genetic studies in lowlanders have shown that individuals with increased HO-1 expression are less prone to pathologies, such as diabetes, atherosclerosis, chronic obstructive pulmonary disease (COPD), and arthritis (Motterlini and Otterbein, 2010). Whether these levels contribute to the variability in pathologies associated with excessive erythrocytosis and chronic mountain sickness in Andeans remain to be determined.

## Clinical Insights

**Figure 2:**
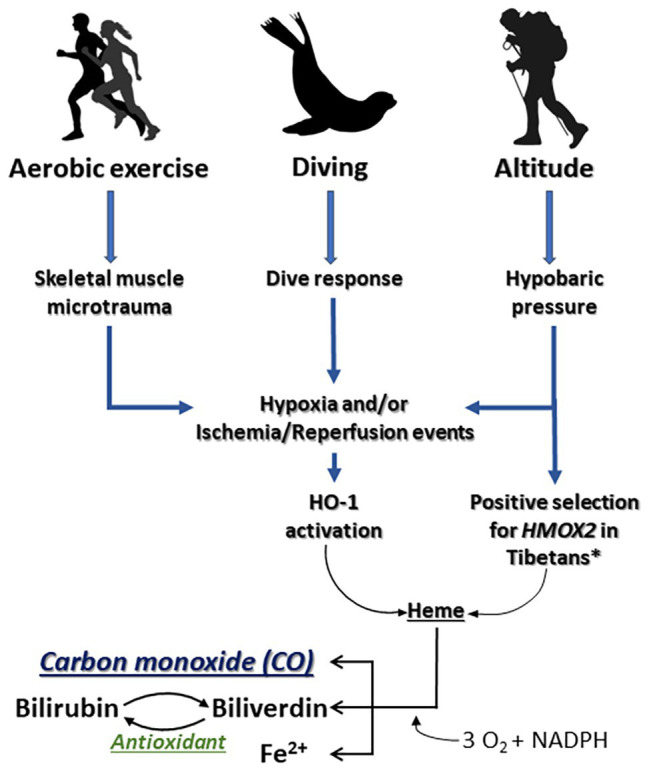
Impacts of aerobic exercise, breath-hold diving, and exposure to altitude on the HO/CO pathway. The stress response protein heme oxygenase-1 (HO-1) responds to intense aerobic exercise and/or skeletal muscle microtrauma by (1) degrading heme that is released during tissue injury as a danger associated molecular pattern and (2) generating bioactive products that contribute to cell and tissue recovery. *Simonson et al., 2010.

Hypoxemia and I/R events are complications inherent to common disease states that often lead to a suite of downstream problems, including adverse cardiopulmonary effects, inflammation, and tissue death. Induction of a battery of stress response genes, such as *HMOX1*, respond metabolically to (1) degrade elevated heme released during tissue damage in part due to an increase in intracellular hemoproteins and (2) to generate bioactive products to further enhance cell and tissue recovery (Figure 2). Hypoxia and inflammation often occur in tandem during infection or I/R events, whereby the hypoxia-inducible factor (HIF) pathway interacts with NF-κB signaling to coordinate molecular responses, including regulation of *HMOX1* (Lee et al., 1997; Rushworth and O’Connell, 2004).

Why would evolution result in a system that increases CO in the height of hypoxia or an inflammatory sequelae and ongoing stress response that otherwise decreases tissue O_2_ availability? Perhaps permissive hypoxia, driven by several selective O_2_ sensors such as HIF-1α, HIF-2α, and/or mitochondrial oxidases, serves as a mechanism by which cells can dynamically adjust to optimize survival in different environments. In each instance, the availability of O_2_, which we define as that which is permissive or allowed, dictates specific cellular responses that benefit the needs of the tissue. While O_2_ is a requisite cofactor for HO-1 activity, HO-1 is also potently induced by hypoxia; in a somewhat paradoxical manner, this results in the generation of CO which then competes with O_2_ for heme binding sites (Figure 2). This observation has led to speculation that CO creates permissive hypoxia in tissues that serve to modulate cellular energetics and protection (D’Amico et al., 2006).

The effects of HO-1 and CO on tissue protection have been clearly demonstrated in models of I/R injury and many other tissue ischemic pathologies (Wegiel et al., 2014). Due to space constraints, we highlight a few of the seminal findings. Induction of HO-1 or exposure to CO offer dose-dependent anti-inflammatory and cytoprotective effects (Otterbein et al., 2016). The cytoprotective effects of CO were first demonstrated in acute lung injury, and these findings rapidly expanded through the work of numerous laboratories to most models of acute organ injury (Hopper et al., 2018). Administration of exogenous CO or higher HO-1 activity increases the expression of anti-inflammatory cytokines and reduces expression of multiple pro-inflammatory cytokines during acute pulmonary inflammation (Konrad et al., 2016; Zhang et al., 2019). In particular, CO inhibited LPS-induced pro-inflammatory signaling by downregulating NADPH oxidase-dependent reactive oxygen species (ROS) production in macrophages, thereby inhibiting toll-like receptor (TLR) signaling (Nakahira et al., 2006). Additionally, CO can inhibit tumor necrosis factor-induced apoptosis *via* a p38 MAPK-dependent mechanism (Ryter et al., 2007). These beneficial effects may also result in part from CO stabilization of HIF-1α (Chin et al., 2007). This is important considering the impact of pulmonary function in hypoxia-induced diseases at high altitude (Selland et al., 1993) and the consistent hypoxemia and lung collapse seen during deep-dives in marine mammals (McDonald and Ponganis, 2012).

CO is also hypothesized to play a role in the control of breathing *via* peripheral O_2_ sensing in the carotid body, where HO-2 is expressed (Prabhakar, 2012). In the presence of hypoxia, decreased HO-2 activity in glomus cells may reduce CO generation and, since CO inhibits cystathionine-γ-lyase production of the excitatory gasotransmitter H_2_S, decreased CO generation may result in increased sensory activity of the glomus cells. This is intriguing given that the gene *HMOX2* is under evolutionary selection in Tibetan populations, who maintain elevated hypoxic ventilatory responses compared to other high-altitude groups (Beall, 2007).

Organ transplant has been perhaps the most well studied when considering translational potential of the HO/CO pathway. The role of HO-1/CO in the kidney was first described in models of I/R injury, where induction of HO-1 or exposure to CO protected the kidney (Schaaf-Lafontaine and Courtoy, 1986; Blydt-Hansen et al., 2003). This large body of work culminated in the initiation of multiple clinical trials, where exogenous CO has safely been administered to transplant patients^1^. The mechanism by which CO imparts its salutary effects remain incompletely understood, but likely targets include hemoproteins such as mitochondria oxidases and therein the regulation of bioenergetics, ROS formation, and consumption of O_2_ (Schallner and Otterbein, 2015; Otterbein et al., 2016). The beneficial effects of HO-1 and CO in organ injury result from preserving mitochondrial health and would dovetail with the observations observed in diving mammals and populations that live at altitude. It is intriguing to speculate that one mechanism for the cytoprotective effects of CO, in instances of tissue hypoxia, may be that CO permits displacement and redistribution of O_2_ within intracellular stores. The activity of HO-1 is known to increase during hypoxia, increasing CO production, which can alter cellular metabolism; thus, another potential mechanism of CO that plays a role in hypoxia tolerance is slowing or shifting oxidative metabolism to glycolysis through permissive use of O_2_ (Figure 2).

The HO/CO pathway has also been the focus of many reports in models of cardiovascular disease, including heart failure and cardiac arrest (Shih et al., 2011). Cardiac muscle cells deficient in HO-1 accumulate lethal amounts of ROS, and mice that survive with embryonic HO-1 deletion exhibit many deleterious effects (Kapturczak et al., 2004). Furthermore, *Hmox1*^−/−^ mice are highly susceptible to I/R injury and, after hypoxia, these animals show evidence of right ventricular infarction (Yoshida et al., 2001; Liu et al., 2005). During reperfusion in the heart, CO administration can decrease infarct size, reduce apoptosis, and increase inotropy. Although those effects were examined mostly in a cardiovascular system, HO-1 also affects other cell types, including skeletal muscle and the physiologic response to exercise (Kozakowska et al., 2018).

Skeletal muscle comprises almost 40% of total body mass in humans, exhibiting major metabolic activity by contributing up to 30% of the resting metabolic rate in adults (Zurlo et al., 1990). The tissue can respond to numerous environmental and physiological challenges (e.g., hypoxia and I/R events) by changing its phenotypic profile and is one commonality across diving animals and individuals living at altitude (Pette and Staron, 2001; Spangenburg et al., 2008; Pabst et al., 2016). Skeletal muscle contraction during exhaustive exercise generates ROS that can promote oxidative damage to myofibers (Reid, 2001), and it has been suggested that HO-1 can protect against exercise-induced injury (Saxena et al., 2010). HO-1 is normally expressed at very low levels in skeletal muscle, but increases dramatically with exhaustive exercise (Pilegaard et al., 2000). The discovery that CO induces mitochondrial biogenesis through specific signaling pathways has raised the possibility that it contributes to the resolution of skeletal muscle injury during and after episodes of oxidative stress, such as exercise or I/R events (Suliman et al., 2007). Intermittent CO breathing after a single exercise test led to an increase in mitochondrial oxidative stress markers and mitochondrial fusion protein expression indicative of mitochondrial biogenesis in skeletal muscle.

From a clinical perspective, based on high disease prevalence and consequences, there is intense interest in both intermittent hypoxemia (as seen in obstructive sleep apnea-OSA) and sustained hypoxemia (as seen in COPD or at high altitude; Soler et al., 2015; Benjafield et al., 2019). The combination of these stimuli (sustained plus intermittent hypoxemia) occurs in some clinical settings, including overlap syndrome (OSA plus COPD), or in OSA patients at high altitude (Marin et al., 2010). The deleterious effects of hypoxemia are well established (Drager et al., 2015; Umeda et al., 2020), although some literature supports a potential protective role of ischemic preconditioning with more mild levels of hypoxemia (Sánchez-de-la-Torre et al., 2018). Regarding CO, some data support its role as a biomarker in OSA (Kobayashi et al., 2008), although its biological impact in these patients has been debated (Owens et al., 2008). Further translational research is certainly encouraged to apply knowledge regarding hypoxia and CO from comparative biology to the clinical setting.

## Conclusions

The concept that evolutionary mechanisms have optimized the biology and physiology of HO and CO is relevant in the context of hypoxia adaptation. Examples can be observed across species and under varying physiologic and pathophysiologic conditions ranging from diving mammals to individuals living at altitude, as well as physical exercise and in response to tissue injury. Increasing our understanding of the natural activity and regulation of the HO/CO pathway associated with injury avoidance and maintenance of cellular health will contribute valuable knowledge regarding the benefits of tissue-specific, moderate-level CO exposure.

## Data Availability Statement

The raw data supporting the conclusions of this article will be made available by the authors, without undue reservation.

## Ethics Statement

The studies involving human participants were reviewed and approved by UCSD IRB #171772. The patients/participants provided their written informed consent to participate in this study.

## Author Contributions

All authors contributed to the article and approved the submitted version.

### Conflict of Interest

LO is a Scientific Advisor for Hillhurst Biopharmaceuticals. AM is funded by NHLBI. He reports income from Merck and Livanova related to medical education. ResMed provided a philanthropic donation to UC San Diego.

The remaining authors declare that the research was conducted in the absence of any commercial or financial relationships that could be construed as a potential conflict of interest.

## Footnotes

^1^www.clinicaltrials.gov
